# Runoff response to climate change and human activities in a typical karst watershed, SW China

**DOI:** 10.1371/journal.pone.0193073

**Published:** 2018-03-01

**Authors:** Yan Xu, Shijie Wang, Xiaoyong Bai, Dongcai Shu, Yichao Tian

**Affiliations:** 1 State Key Laboratory of Environmental Geochemistry, Institute of Geochemistry, Chinese Academy of Sciences, Guiyang, China; 2 Puding Karst Ecosystem Research Station, Chinese Academy of Sciences, Puding, Guizhou, China; 3 University of Chinese Academy of Sciences, Beijing, China; 4 Guizhou Province Bureau of Hydrology and Water Resources, Guiyang, China; Chinese Academy of Forestry, CHINA

## Abstract

This study aims to reveal the runoff variation characteristics of long time series in a karst region, analyse comprehensively its different driving factors, and estimate quantitatively the contribution rates of climate change and human activities to net runoff variation. Liudong river basin, a typical karst watershed in southwest China, is the study site. Statistical methods, such as linear fitting, the Morlet wavelet analysis, normalized curve and double mass curve, are applied to analyse the runoff of the watershed. Results show that the runoff in the karst watershed during the research period exhibits a three-stage change and the abrupt change points are the years 1981 and 2007: (1) 1968–1980, the runoff initially exhibited a trend of sustained decreasing and then an abrupt fluctuation. The runoff was obviously destroyed through precipitation-producing processes. Improper land utilisation and serious forest and grass destruction intensified the fluctuation variation amplitude of the runoff. (2) 1981–2006, the changing processes of runoff and precipitation exhibited good synchronism. Precipitation significantly affected runoff variation and human activities had a slight interference degree. (3) 2007–2013, the fluctuation range of runoff was considerably smaller than that of precipitation. The significant growth of forest and grassland areas and the increase in water consumption mitigated runoff fluctuation and greatly diminished runoff variation amplitude. According to calculation, the relative contribution rates of precipitation and human activities to net runoff variation with 1981–2007 as the reference period were −81% and 181% in average, respectively, during 1968–1980, and −117% and 217% in average, respectively, during 2007–2013. In general, the analysis of runoff variation trend and of the contribution rate of its main influencing factors in the typical karst watershed for nearly half a century may be significant to solve the drought problem in the karst region and for the sustainable development of the drainage basin.

## 1. Introduction

In recent years, the runoff formation in a changing environment has become an important scientific problem in hydrology [1–4]. A better understanding of the runoff changes and their potential driving forces are thus of paramount importance to effectively utilize water resources and reasonably manage river flows. Generally, climate change and human activities are significant factors influencing runoff variation [5–7]. And these factors affect the hydrology and water cycle in a region [8–13]. In a long-term span, climate change is a dominant factor affecting runoff in drainage basins. In a short-term span, human activity interference is the main cause of hydrology process variation in a drainage basin [14,15].

Recently, extensive studies have been conducted on the response of runoff variation to climate change and human activities of different rivers in different areas worldwide. Moreover, separation and quantification of the two driving factors on runoff variation have become topical issues in hydrology [16]. In the arid regions, for example, much attention has been paid to distinguishing the contributions of climate change and human activities to the changes in runoff over the past half-century, in order to provide effective control measures to protect the fragile local ecosystem and ensure the sustainable development of the Loess Plateau water resources [17,18]; in the semiarid regions, several previous studies have qualitatively investigated the influence of climate change and human activities on runoff in the Yellow River [19]. And human activities have been shown to be the main cause of the decrease in water discharge [20]; in the humid regions, the annual water and sediment discharge exhibited a decreasing trend over the last decades in the Yangtze River. This reduction in water discharge is mainly controlled by precipitation change (72%), while human activities contributed 86% of the reduction in sediment discharge [21]. At present, experimental watersheds methods and physical mechanisms-based hydrological models have been widely used to quantitatively estimate the response of runoff to climate change and human activity interference [7,22]. Experimental watersheds methods played an important role in water circulation process research [23]. For example, Wang et al. (2015) determined the impact of human activities within paired datasets under the same or similar weather conditions (SWC) [24]. However, experimental watersheds methods are purely empirical methods based on watershed-scale experiments, which should be long-term and continuous. And these methods are also applicable only to small research regions [25]. Meanwhile, some hydrology models based on the physical mechanism have been used to study the hydrological process and its response to interference through the development of computer and remote sensing technology. This type of model includes soil and water assessment tool [26], precipitation runoff modelling system [27,28]and land use change on hydrology by ensemble modeling [29,30]. For example, Chang et al. (2015) employed the Variable Infiltration capacity (VIC) hydrological model to distinguish the impacts of climate variability and human activity on hydrology in the Weihe River Basin, and suggested that the percentages in change of runoff caused by human activity were 64%, 72%, 47%, and 90% in the 1970s, 1980s 1990s and 2000s, respectively [31]. Depeng Zuo et al. (2015) discussed the impacts of land-use and climate changes on water and sediment yields in the Huangfuchuan River basin of the Loess Plateau by combined use of statistical tests, SWAT and land-use maps. The result indicated that the Grain for Green Program has a significant effect on the changes [32]. Under usual circumstances, however, model factor parameterization is challenging in various methods and calibration methods because a large quantity of data required for simulation analysis with a hydrophysical model is not available. In addition, the practical application of a physical model is largely limited because of the particularity and complexity of the geological structure in karst drainage basins even though such a model exhibits a clear physical mechanism and high simulation accuracy [11,33,34]. As an alternative, statistical methods have been proven effective in discussing the response of runoff to interference of various factors. Kong et al. (2016) investigated the features of variations in runoff increment in the YRB and quantitatively evaluated the impact of climate change and human activities on the mean annual net runoff by residual analysis based on double mass curves (RA-DMC) [35]. Xu (2011) used linear regression to separate and quantify the two driving forces on annual runoff variations, and showed that 78.6% of annual observed runoff and 72.9% of natural runoff decreases could be explained by climate change [2]. Zhang et al. (2014) [7] applied a non-linear relationship exists between runoff and the climatic system in a ‘reference stage’, then assessed the respective impact of human activities and precipitation on runoff in the ‘affected stage’. However, previous studies focused mainly on the non-karst areas and the impact of climate change and human activities on runoff change is not yet fully understood in karst regions.

The karst watershed of southwestern China is located in the upper reaches of Yangtze River and Pearl River in China. And it has both the ecological and environmental significance for the survival and development of about 100 million people. However, the rock is fragile, the soil is shallow, the rain is likely to leach into the ground, the rocky desertification is serious and the ecological environment is fragile in the karst area because of the unique geological background and features of the environment [36,37]. Serious functional shortage of water and seasonal drought problems exist in karst regions [38–40]. In addition, serious soil and water losses are caused by improper human activities. Agriculture production and human life are seriously threatened [41]. Water problems have been a key factor restricting the development of social and economic environment in karst regions of southwest China. So the research on the water cycle in karst watershed is significant for the environmental protection and sustainable development of the area.

In karst area, many scholars have analyzed the evolution characteristics of precipitation and runoff series. Shi et al. [42] analyzed the annual and seasonal variation of runoff process in Guizhou karst watershed based on L-moments method and MK method. Chen et al. [43] used BP ANN to establish runoff prediction model of surface karst spring in Luota River area, Hunan Province. And on this basis, some scholars have qualitatively discussed the driving factors of the runoff process. For example, some of them [44–46] discussed the rainfall–runoff characteristics on the slope of different land use types in a typical karst region based on the observation of runoff plots and obtained varying results due to different geological backgrounds and vegetation types. Kong et al. [47] have qualitatively discussed the influence of human activities on runoff in karst watershed based on the analysis of the variation characteristics of runoff series in the past 50 years, and indicated that human activities generally increased the runoff. Furthermore, some scholars tried to apply hydrological models those are appropriate in non-karst regions to karst regions, and quantitatively discussed the water cycle in karst regions. For instance, in order to assess the sustainability of the actual water use in the Island of Crete, Anna Malagò et al. (2016) developed a methodology combining the SWAT model and a karst-flow model [48]. However, an in-depth discussion of the influence of natural factors and human activities on runoff variation in a typical karst drainage basin is currently lacking. Moreover, how to evaluate the influence of climate change and human activities is still an open question which needs further research to provide a comprehensive and reasonable explanation to the observed runoff variation.

Based on data from Liudong river basin, statistical methods are applied to analyse the runoff of the watershed. The purposes of this study are: (1) to reveal the time series evolution characteristics of runoff and precipitation in the research region in the recent 50 years; (2) to analyse comprehensively the driving factor of runoff variation; and (3) to estimate quantitatively the contribution rate of precipitation and human activities to runoff variation. The present work will provide a better understanding of the interactions between human and nature. Meanwhile, provide important insights into water resources management in the karst area.

## 2. Data and methods

### 2.1. Study area

**Fig 1:**
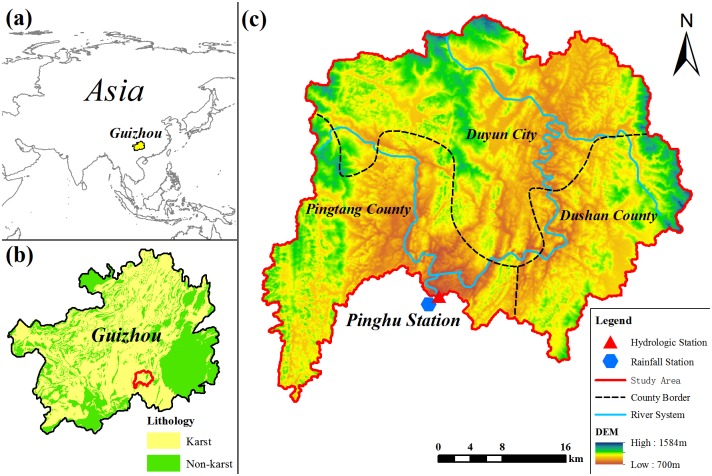
Location map of the study area. Fig 1 (a) and (b) show the location of research area in the maps of Asia and Guiyang Province, respectively. For Fig 1(a), yellow area shows location of Guizhou. For Fig 1(b), red boundary shows research watershed selected. Yellow area represents karst area and green area represents non-karst area. For Fig 1(c), hydrologic station (red triangle), rainfall station (blue polygons), study area (red line), county border (black dotted line), and river system (blue line) in the study area are presented. And Digital Elevation Model (DEM) also shows on the map. Maps in Fig 1 were generated by ArcGIS 10.2 using the free download data online of the Geospatial Data Cloud (http://www.gscloud.cn/) and URL link of the software is http://www.esri.com/.

The specific research region selected in the article is the watershed controlled by Pinghu Hydrological Station of Liudong river. This watershed is located in Qiannan of southern Guizhou, China. It also belongs to the Hongshui river branch, Xijiang river system of Zhujiang river drainage basin between 107°06′–107°38′ E and 25°43′–26°09′ N. The main stream of the drainage basin originates from Baba, Laling township, Dushan county, and it flows through Duyun, Pingtang and Dushan counties. The drainage basin is 53km long and 49km wide, and its total area is 1493 km^2^. Fig 1 shows the distribution of water systems and stations in the drainage basin. The topography is characterised by higher in the north and lower in the south. The average elevation is 1020 m. Emergence stratums are Carboniferous, Permian, and Triassic strata. Carbonate rocks are widely distributed, which is a typical karst terrain featured with karst depression, funnel, sinkhole and underground river development. The climate in the drainage basin is subtropical humid monsoon climate. The annual mean temperature is 17°C, and average annual rainfall is 1220mm. The land use types are mainly forest land, cultivated land and shrub. The vegetation is mainly composed of masson pine, firewood forest and clastic rock bush wood, and the forest coverage rate is approximately 50%.

### 2.2. Materials and methods

**Fig 2:**
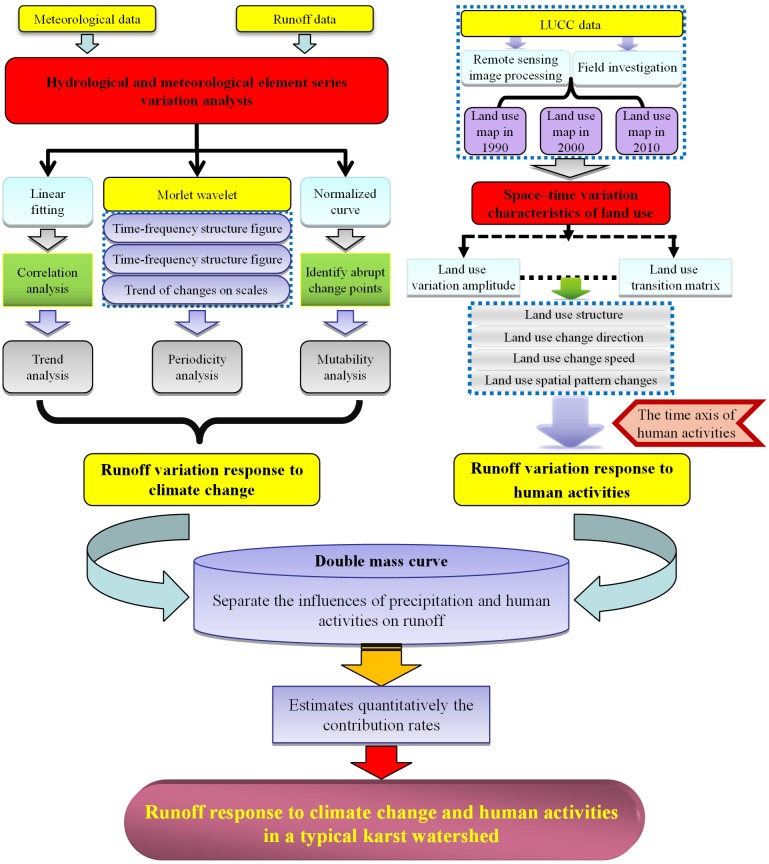
The technology roadmap of the whole article.

Runoff data adopted in this article is the monthly runoff data from 1968 to 2013 of the controlling surface water hydrological station—Pinghu Hydrological Station—in the midstream and upstream of Liudong River drainage basin. These runoff data are obtained from the Hydrology and Water Resources Bureau of Guizhou Province. The daily precipitation data and the daily mean temperature data from 1968 to 2013 of Pingtan Station provided by the Website of China Meteorological Data Service Center (CMDC) (http://data.cma.cn/) are selected as meteorological data. Remote sensing data are obtained from U.S. Geological Survey (http://glovis.usgs.gov/). The three sets of TM images (1990, 2000 and 2010) are obtained, and their resolutions are 30 m. The quality of images meets the application requirements with a low cloud cover (2.32%, 0.19% and 0% respectively). In consideration of the geographic characteristics, image quality and the research objective of this article, the following classification system is adopted: forestry, grass land, water area, arid land, paddy field and building lot. All the image data in this research are adopted standard false colour composite images, and they are combined with Google Earth high-resolution images. The visual interpretation is provided in human–machine interface mode, and the images are treated after classification. Overall accuracy values are 96.89%, 98.55% and 93.17% respectively, and kappa coefficient values are 0.95, 0.97 and 0.93respectively. The interpretation results were validated and modified through field investigation, which derives the three-phase land use vector diagram in 1990, 2000 and 2010. The data processing software programs are MATLAB 7.0, SPSS 22.0, ENVI 5.2 and ArcGIS 10.2. The technology roadmap of this research is shown in the Fig 2.

#### 2.2.1. Land use variation analysis

In this article, the land use variation of the drainage basin in the research period is obtained from remote sensing image data analysis and historical literature recordation to analyse further the influence of the underlying surface caused by human activities on runoffs.

The space-time variation characteristics of land use are revealed by two indices: land use variation amplitude and land use transition matrix. The specific method is as follows:

1. Land use variation amplitude:
  $$P_{i}=\left(LU_{i1}-LU_{i0}\right)/LU_{i0}\times 100\%$$ (1)
  where *LU*_*i*0_ and *LU*_*i*1_ represent the area at the beginning of research, and the area at the end of research of *i*th land use type in the research region, respectively.
2. Land use transition matrix:
  The transition probability matrix amongst land use types in different periods is established, by which the transition rate of one type of land use to other land use type during a certain period is analysed directly with the size of factor for land type corresponding to this matrix. The mathematical formula of the transition matrix can be expressed as:
  $$P=\left[\begin{array}{cccc} p_{11} & p_{12} & \cdots & p_{1n}\\ p_{21} & p_{22} & \cdots & p_{2n}\\ \vdots & \vdots & \ddots & \vdots \\ p_{n1} & p_{n2} & \cdots & p_{nn} \end{array}\right]$$ (2)
  where *P* represents the area, *n* represents the land use type, and *i* and *j* in *p*_*ij*_ represent the land use types at the beginning and end of research, respectively.

#### 2.2.2. Hydrological and meteorological element trends and periodic variation analysis

Based on the datum of runoff and precipitation in the target region, the trend analysis is conducted statistically and the periodic variation research is conducted with Morlet wavelet analysis.

Wavelet analysis is a superior method for the study of time series variation. It is a time-frequency multi-resolution method which reflects the partial variation characteristics of time series and identifies the evolution characteristics of time series in different scales.

The wavelet function Ψ(*t*) refers to function which is oscillating and can decrease abruptly to zero. If its Fourier transform, then Ψ(*ω*) meets the condition:

$$\int_{R}\frac{{\left|\Psi \left(\omega \right)\right|}^{2}}{\left|\omega \right|}d\omega <\infty$$ (3)

Where Ψ(*t*) is the basic wavelet or mother wavelet. A group of functions is derived through the magnification and translation of Ψ(*t*):

$$\Psi_{a,b}\left(t\right)=\frac{1}{\sqrt{\left|a\right|}}\varphi \left(\frac{t-b}{a}\right)$$ (4)

where Ψ*a*,*b*(*t*) is the continuous wavelet, *a* is the scale factor, and *b* is the time factor.

In practical applications, the continuous wavelet is usually discrete: *a = aj 0*, *b = kb*_*0*_, where *aj 0>*1, *b*_*0*_*∈*R, and *k*, *j* are integrals. The discrete wavelet transition of *f(t)* is as follows:

$$W_{\Psi }f\left(j,k\right)=a_{0}{}^{-j/2}\int_{R}f\left(t\right)\Psi \left(a_{0}{}^{-j}t-kb_{0}\right)dt$$ (5)

where $W_{\Psi }f\left(j,k\right)$ is the wavelet coefficient, which is the degree of approximation of the signal and wavelet in this part.

Morlet wavelet has a similar waveform to runoff time series and good localisation in the time frequency domain. Therefore, Morlet complex wavelet is selected in this article to conduct continuous wavelet transform to annual runoff series in the research region and to extract the wavelet coefficient and the wavelet variance of the series. Wavelet variance reflects the distribution of fluctuation energy with scale and can be used to determine the main time scale in a time series for the peak of variance corresponds to different primary periods of series from large to small. The horizontal section of wavelet coefficient indicates the change of wavelet of each period in the time series with time. The wavelet coefficient has a positive relationship with runoff, and the alternates of maximum and minimum values of wavelet coefficient under a certain time scale correspond to high and low flow cycles in this scale; thus, the multi-time scale periodicity and discontinuity of time series can be identified by analysing wavelet coefficient.

#### 2.2.3. Catastrophe analysis of hydrological and meteorological elements

Climate change affects runoff generation process in the setting of changing human activity. This can be studied by comparison of runoff generation between “base-line” and “measure” periods. The dividing point between these two periods can be determined by time series analysis, to detect the abrupt change in the temporal variation in runoff.

The standard value *(K-1)/C*_*v*_ is used in representing the multiyear variation of runoff and precipitation and in identifying the abrupt change points of runoff to eliminate the influences of annual runoff, precipitation unit, dimensional differences and coefficient of variation (*C*_*v*_) and the differences of unit and dimension in this article. The specific method is as follows:

$$K=X_{i}/\bar{X}$$ (6)

$$C_{v}=\sigma /\bar{X}$$ (7)

$$\sigma =\sqrt{\sum_{i=1}^{n}{\left(X_{i}-\bar{X}\right)}^{2}/\left(n-1\right)}$$ (8)

where *X*_*i*_ is precipitation or runoff in *i*th year, $\bar{X}$ is multiyear average precipitation, and *R*_*i*_ is the precipitation or runoff in *i*th year.

#### 2.2.4. Estimating the relative impact of climate change and human activities on runoff

Multiple regression is usually used to determine the contribution rate of natural factors and human activities to runoff variation. However, the research results obtained about the same research object by different researchers have large differences because the weight assignment of each influencing factor is determined by human. The multiple correlation coefficient between each factor of multiple regression is small; thus, the obtained result accuracy is also small. This finding shows that this method is considerably limited [49]. Thus, the double mass curve method is adopted in this article to analyse quantitatively the influence of climate change and human activities on runoff. The contribution rate of human activities and climate change to runoff is derived by establishing the rainfall–runoff model when the influence of human activities is small and by restoring the runoff when the influence of human activities is large. Both independent and dependent variables are cumulants, thereby eliminating the influence of the annual fluctuation of measured data to some extent and creating conditions for further quantitative analysis. The specific method is as follows [50]:

On the basis of the time series analysis of runoff and precipitation, the runoff time series is divided into the reference section, on which the influence of human activities is small, and the variation section, on which the influence of human activities is large.

1. The following relation is derived by conducting linear regression to cumulative precipitation ∑P and cumulative runoff ∑R in the reference section:
  ∑P=k∑R+b (9)
2. The relation of (9) is applied in the variation section, and cumulative runoff is calculated by regarding cumulative precipitation in variation section as X. This result may be taken as the runoff of the second period in the supposition that no human actions were implemented.
3. The differences between the measured annual runoff and calculated runoff in the two sections are derived by deriving the annual runoff from the calculated cumulative runoff in the variation section. The difference is the runoff variation caused by human activity interference and climate change:
  δ=HRva−Rvc (10)
  δ=CRvc−Rra (11)
  where *δ*_*H*_ and *δ*_*C*_ are the runoff variations caused by human activities and climate change, respectively. *R*_*va*_, *R*_*vc*_ and *R*_*ra*_ are the observed runoff in the variation section, the calculated runoff in the variation section and the observed runoff in the reference section, respectively.
4. The contribution rate is adopted to quantify its influence degree by indicating the influence of human activity change and climate change on runoff variation during each period. The following is the calculation formula:
  $$Q_{g}=\frac{\delta_{H}}{\Delta R}\times 100\%\;\text{or}\;Q_{g}=\frac{\delta_{C}}{\Delta R}\times 100\%$$ (12)
  where *Q*_*g*_ *i*s the contribution rate (%) of human activity interference and climate change to runoff evolution, and Δ*R* is the runoff difference between the reference and variation sections.

It is worth noting that we focus on the contribution of human activities and climate change to the net runoff component in this study. The combined effect of the two factors is assumed as the unit. On the one hand, if both elements cause the runoff to change in the same direction, that is, decrease or increase at the same time, *Q*_*g*_ is less than one. On the other hand, when the impact of climate change on the runoff is opposite to that of human activities, the absolute value of the contribution rate of the two factors maybe larger than 1. For example, when the value of net runoff is positive, but climate change has a negative impact on runoff, then the contribution rate of human activities to runoff variation will be greater than +1.

## 3. Results and discussion

### 3.1. Analysis of runoff variation response to climate change

The principal climatic factors affecting runoff variation are precipitation and temperature. Precipitation change will directly disturb the discharge of the drainage basin, whereas temperature change influences the runoff by altering the capacity of evaporation. The following discussion analysed from temperature and precipitation respectively.

#### 3.1.1. Correlation analysis of temperature and runoff

**Fig 3:**
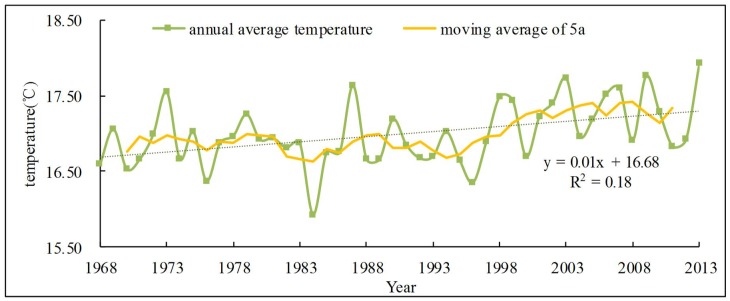
The linear trend of annual temperature. In the figure, green and yellow lines with markers respectively represent annual average temperature and the moving average of 5a of temperature. And green dotted line is the trendline of annual average temperature.

**Fig 4:**
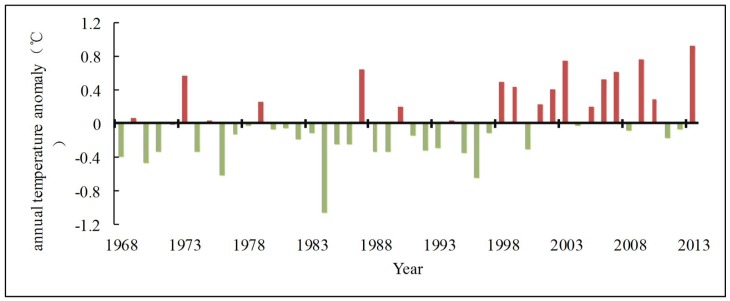
Anomalous variation of temperature. In the figure, red and gray bars respectively represent positive and negative departures from average value.

The annual temperature change curve (Fig 3) shows that the average temperature does not show significant growth trend in the past 50 years. The linear change rate is 0.1°C/10a, which does not reach 90% significant level. The highest mean annual temperature was 17.93°C (2013) and the lowest was 15.93°C (1984). The average temperature was 16.99°C during the study period. The temperature variation remained relatively stable and the relative change range was only 1.13. It can be seen from the 5-year average sliding curve (Fig 3) and the anomaly map (Fig 4) that the temperature in the basin was stable during the 1970s and had no obvious change, and then shown a visible downward tendency. The annual average temperature was continuously lower than the average level until the middle 1990s. Subsequently, the temperature began to increase significantly. The negative anomaly was dominated before 1997, and then the positive anomaly followed. It is consistent with the background that there was a cold period before the 1990 s and a warm period later in China.

**Fig 5:**
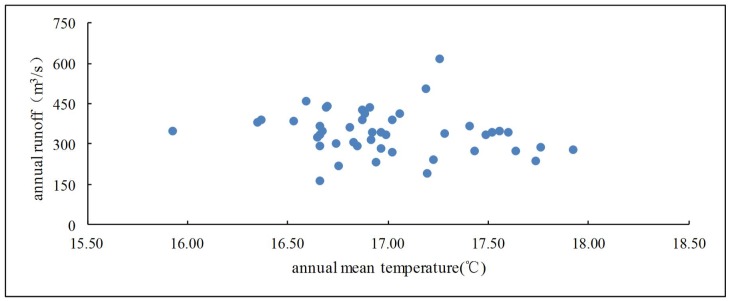
Scatterplot of mean annual temperature and annual runoff.

The relationship between annual mean temperature and annual runoff is analyzed by plotting the scattergram (Fig 5). It can be seen that there is no obvious statistical relationship between temperature and runoff over the past 50 years. In order to further analyze the relationship between temperature and runoff in the study area, SPSS was utilized to analyze the correlation between them. The result shows that the correlation coefficient between annual runoff and annual mean temperature was -0.18, but the correlation does not prove a statistical significance (P-Value was 0.23). Therefore, there was no correlation between temperature and runoff in the study area. This is consistent with previously studies in other typical karst areas of southwestern China [44,51,52]. Furthermore, it implied that evaporation has little effect on runoff variation. Meanwhile, the effect of temperature on runoff is much smaller than that of precipitation and other human activities due to the small temperature change in the short term.

#### 3.1.2. Correlation analysis of precipitation and runoff

**Fig 6:**
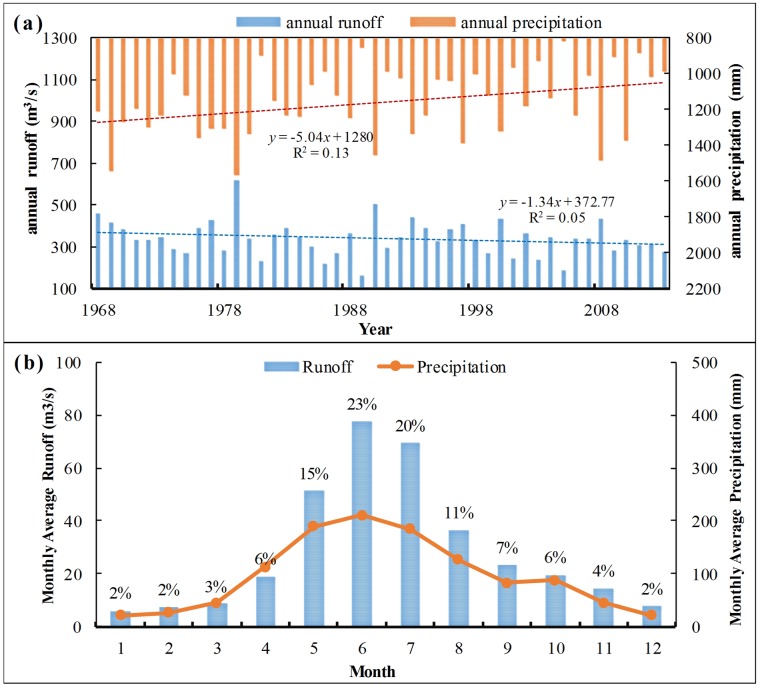
The interannual variations of runoff and precipitation in 46 years (a) and the annual variation curve of the average runoff and precipitation for several years (b). In Fig 6(a), blue and red bars respectively represent runoff and precipitation. And blue dotted line, red dotted line are respectively the trendline of them. In Fig 6(b), blue bars and red line respectively show the annual distribution of runoff and precipitation, and only the percentage of monthly runoff are showed in the picture.

For the recent 50a, the interannual variation of runoff and precipitation in the drainage basin is shown in Fig 6(a). Except for the interannual normal fluctuation, the annual runoff shows a decreasing trend with a variation rate of −1.34 m^3^/s·a, which does not reach 90% significant level. High and low flow values appeared in 1979 and 1989. The difference between high and low flow is 37.97 m^3^/s, and the annual extreme value ratio is 3.8. The precipitation shows a decreasing trend. The climate tendency rate is −5.04 mm/a, and the decreasing trend is significant at the α = 0.05 significant level. The annual variation curve of the average runoff and precipitation for several years in Liudong river drainage basin given in Fig 6(b) shows that the runoff changes with the precipitation and that the annual runoff process line basically corresponds with the precipitation process line, which shows a single peak type. The runoff in January is the minimum value of the whole year, and the annual average value is only 6.15 m^3^/s. The runoff reaches the maximum value in June, and its annual average value is 77.51 m^3^/s. The distribution of runoff during the year is not even, and it concentrates in flood period and counts 69% of the annual average runoff.

**Fig 7:**
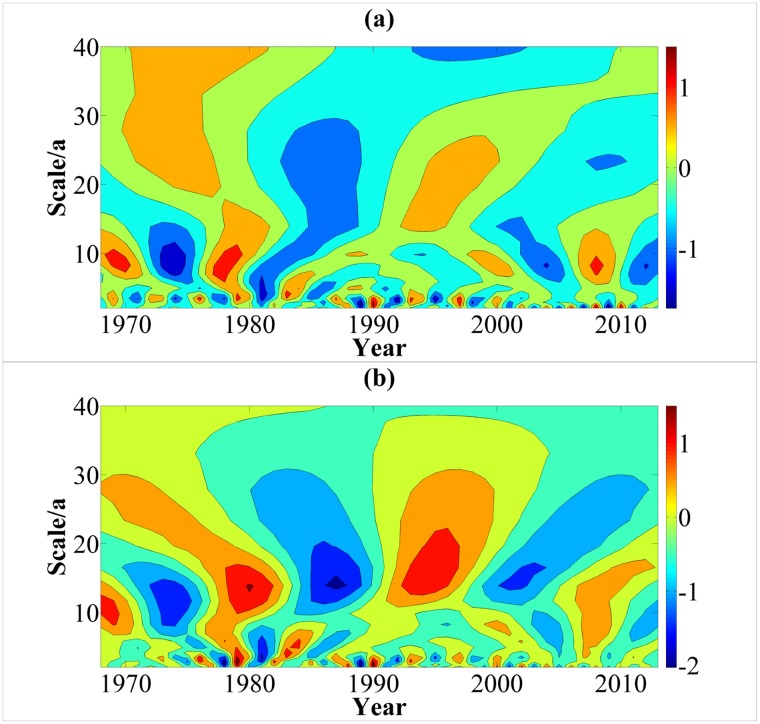
Time-frequency structure figure of the real part of Morlet wavelet transform coefficients of annual precipitation (a) and runoff (b). Fig 7 describe the high and low flow phase structures under different time scales by employing Matlab 7.0. And tone from warm to cold represent high values to low values of the real part of Morlet wavelet transform coefficients.

As shown in Fig 6, the trend of runoff is largely consistent with the precipitation variation in the study area. But the decrease of annual precipitation is larger than that of the annual runoff. The correlation coefficient between annual runoff and precipitation is 0.799, and the significance level is less than 0.001, indicating that there is a high correlation between them. These mean that the evolution of runoff in the study area is mainly affected by precipitation. This is consistent with previous studies on karst areas in Southwest China [53]. Fig 7(a) and 7(b), which illustrate time-frequency representation of the real part of wavelet coefficient for precipitation and runoff, show the high and low flow phase structures under different time scales. Diagrams indicate that the variations of runoff and precipitation for the recent 50a in the research region are superimposed by periodic oscillations of different lengths. The high and low flow cycles of precipitation and runoff corresponding to different time scales are different. And the high and low flow cycles of the small scale are included in the complicated high and low flow structures of a large time scale. In the interdecadal scale, the 8-12a scale signal is the most obvious for precipitation variation. In addition, precipitation has a minor cycle of 20-25a scale, but the isoline is loose. The closing state of isoline is not good, and the signal is weak. However, for the interdecadal period of runoff, the 10-20a scale signal is the most obvious. Its central time scale is approximately 14a. For annual variation, the periodic variations of precipitation and runoff have the strongest signal in 2-4a scale. The positive and negative phases appear alternatively and are distributed intensively. The closing state of isoline is good and has high and low frequency oscillations of wavelet coefficient.

**Fig 8:**
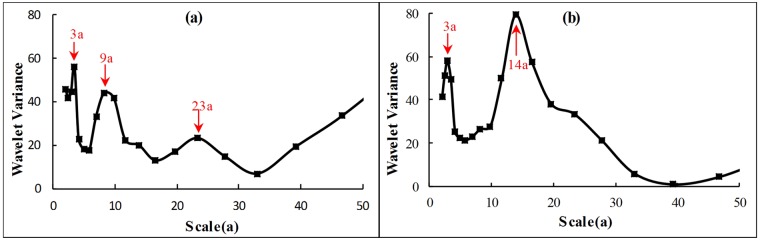
Wavelet variance diagram of annual precipitation (a) and runoff (b). In figures, black lines paint the fluctuation of wavelet variance with scale. Red numbers and arrows point out the peak of variance which corresponds to different primary periods of series from large to small.

The wavelet variance diagram (Fig 8) is plotted to verify further the precipitation and runoff periods. Fig 8(a) shows that precipitation has three peak values, namely, 3a, 9a and 23a. The first main period is 3a, and it has the strongest periodic oscillation. Then, the main periods 9a and 23a follow. Whereas, two obvious peak values exist in the wavelet variance diagram of runoff (Fig 8(b)), and these peak values correspond to time scales 3a and 14a. The first peak value corresponds to time scale 14a, and the periodic oscillation of this time scale is the strongest. This period is the first main period of the drainage basin, and 3a is the second period.

**Fig 9:**
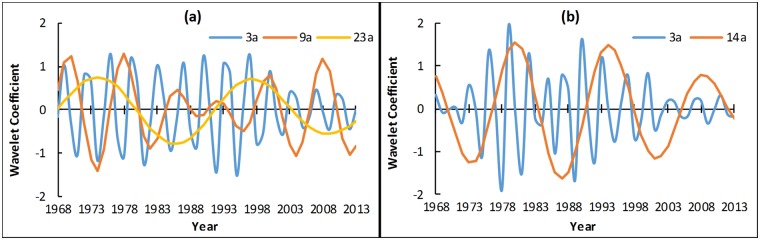
Trend of changes in annual precipitation (a) and runoff (b) on scales of major periods. For Fig 9(a), blue, red and yellow lines respectively represent the main periods 3a, 9a and 23a. And for Fig 9(b), blue and red lines respectively represent the main periods 3a and 14a. The main periods were got from the verification result of wavelet variance of Fig 8.

According to the verification result of wavelet variance, the wavelet coefficient diagram of each primary period (Fig 9) is plotted. For precipitation, two oscillations analysed from 23a scale. These oscillations are shown in detail: high flows in 1968–1979 and 1992–2003; low flows in 1980–1991 and 2004–2013. The periodic variation of 9a scale is mainly active in the 1990s and at the beginning of the 20th century. Whereas, the periodic variation of 3a scale is stable throughout the analysis period of precipitation and applicable to the overall situation. As analysed from 14a scale, the runoff variation experiences three high and low flows for the runoff. The periods 1968–1969, 1977–1983, 1991–1997 and 2006–2012 are positive phases, that is the abundant stage. Other time periods are dry. The periodic variation of 14a scale is stable throughout the analysis period and applicable to the overall situation. Its periodic variation energy is the largest, and it has the largest contribution to the variance of the original runoff series. The runoff fluctuation of 3a time scale is large and this time scale has many abrupt change points. The periodic variation is mainly active during 1970–1990. In general, the runoff in 14a scale will enter a dry period for a certain period in the future. On the contrary, the runoff in 3a scale will enter an abundant period. The reason is that the abrupt change points in a small time scale gradually regress to normal points with the increase in time scale, however, singular points increase with the decrease in time scale. Some normal states in a large time scale are catastrophe states in a low level. Therefore, the target region is in dry period from a large time scale.

Current studies suggested that the periodic variation of meteorological and hydrological series was concerned with the motion of celestial bodies and solar activity [54]. The period of runoff for 14a and precipitation for 9a and 23a coincide with the Sunspot period of about 11a and the Haier cycle of about 22a. The period of runoff and precipitation for 3a is related to the quasi-periodic for 5–7 year period of ENSO event [55]. Those indicate that the precipitation and runoff were much affected by solar activity and global climate change.

**Fig 10:**
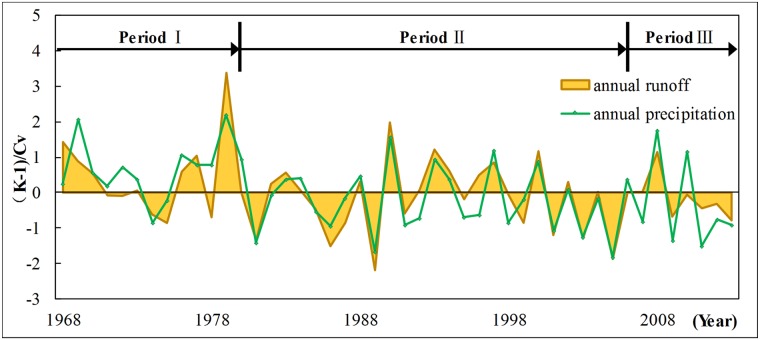
Normalized curve of annual runoff and annual precipitation. This figure shows the multiyear variation curve after standardization treatment of annual runoff (yellow area) and annual precipitation (green line) in study area. Time periods 1968–1980, 1981–2006 and 2007–2013 are represented by period I, II and III. Period divided on the basis of the goodness of fit between annual runoff and annual precipitation. Annual runoff and precipitation exhibit a good synchronous change relationship in the fluctuation in period II and show a clear separation in period I and III.

The contrastive analysis of the time-frequency distribution diagram of runoff and precipitation shows that the variation trends of runoff and precipitation in the research region are generally similar in the time and frequency domains. In addition, variation periods are synchronised to some extent, which is consistent with the aforementioned results. The results reaffirmed that the variation of precipitation is the main cause of runoff variation to some extent. However, an obvious difference also exists amongst their periodic variations. The precipitation series has 9a obvious period, whereas runoff has 14a obvious period, which is slightly larger than the variation period of precipitation. Moreover, runoff has no obvious period variation in 23a scale. In 3a scale, the precipitation series is stable throughout the analysis period. However, the runoff series is only active from the end of 1970 to 1990. This observation indicates that the periodic variation of the remaining time periods is largely influenced by nonclimate factors. Therefore, the runoff is influenced by climate change and largely influenced by human activities. Under different time scales, different variation combinations of climate change and human activities exert different influences on runoff. Fig 10 shows the multiyear variation curve after standardization treatment of annual runoff and annual precipitation in Liudong river basin. The figure shows that the annual runoff and annual precipitation from 1981 to 2006 exhibit a good synchronous change relationship in the fluctuation, but those before 1981 and after 2006 show a clear separation. From 1968 to 1973, the precipitation shows fluctuation and changes, whereas the runoff shows a continuous decreasing trend. From 1978 to 1980, the runoff shows abrupt fluctuation which is far off the precipitation variation curve. From 2007 to 2013, the fluctuation amplitude of the runoff in the research region is considerably smaller than that of precipitation. Relevant studies [56,57] have shown that precipitation variation may be magnified in runoff. However, the runoff fluctuation amplitude from 1968 to 1973 and from 2007 to 2013 in the research region is considerably smaller than precipitation, meanwhile the runoff fluctuation variation from 1968 to 1979 and from 2007 to 2013 is not completely synchronised with precipitation variation. This finding indicates that the precipitation–runoff relationship during these two periods is interfered by non-precipitation factors, such as land cover change and industrial and agricultural water use. This finding also proves the preceding research results.

According to the preceding analysis, time periods 1968–1980, 1981–2006 and 2007–2013 are represented by I, II and III, respectively. A list is provided to compare the average value, standard deviation and *Cv* (Table 1) of precipitation and runoff during time periods I, II and III. During time period I, both the standard deviation and coefficient of variation for annual average precipitation are smaller than those during time period II. However, the standard deviation and coefficient of variation for runoff remain the same as those during time period II. The ratio of coefficient of variation for runoff to that of precipitation during the time period I is 2, which is smaller than 1.67 during time period II. Therefore, compared with that during the time period II, the human activity interference during time period I intensifies the fluctuation variation amplitude of the runoff. During time period III, the standard deviation of precipitation increases by 71.2mm, and the coefficient of variation increases by 0.07 compared with those during time period II. However, the standard deviation of the runoff decreases by 29.7mm, and the coefficient of variation decreases by 0.09 compared with those during time period II. The ratio of coefficient of variation for runoff to that of precipitation during time period III is 0.73, which is considerably smaller than that during time period II. Therefore, unlike those during time period II, the human activities during time period III significantly influence runoff variation and mitigate runoff fluctuation amplitude.

**Table 1:** Variations of annual streamflow and precipitation in Liudong River watershed.

	1968–1980 (period I)				1981–2006 (period II)				2007–2013 (period III)			
	Mean	Standard Deviation	Cv	Ratio of Cv	Mean	Standard Deviation	Cv	Ratio of Cv	Mean	Standard Deviation	Cv	Ratio of Cv
Runoff (m^3^/s)	376.56	92.04	0.24	2.00	327.16	82.57	0.25	1.67	328.33	52.87	0.16	0.73		
Precipitation(mm)	1288.02	152.28	0.12		1115.82	165.33	0.15		1096.67	236.53	0.22			

According to the preceding analysis, the runoff in the drainage basin in the research region shows a three-phase variation. The years 1981 and 2007 are taken as the dividing points, and the period 1981–2006 is regarded as reference section with the periods 1968–1980 and 2007–2013 are regarded as variation section, respectively. In the reference section, the fluctuation of the runoff in the drainage basin is mainly caused by climate change, and precipitation exerts a decisive influence on runoff variation. However, in the variation section, the variation of the runoff in the research region is caused by both climate change and human activities.

### 3.2. Analysis of runoff variation response to human activities

#### 3.2.1. Changes in land cover

**Fig 11:**
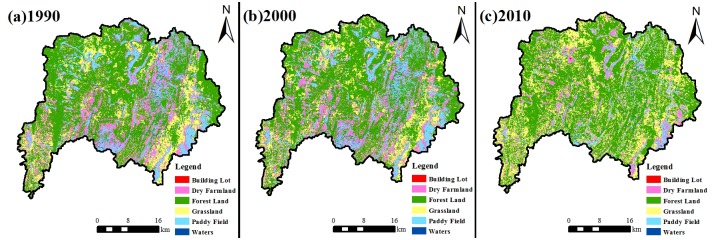
Spatial distribution maps of land use in 1990 (a), 2000 (b) and 2010 (c). Forestry, grass land, water area, arid land, paddy field and building lot were classified in maps. Overall accuracy values of three-phase land use vector diagram are 96.89%, 98.55% and 93.17%, and kappa coefficient values are 0.95, 0.97 and 0.93. Maps in Fig 11 were generated by ArcGIS 10.2 and ENVI 5.2 and the URL links of software are http://www.esri.com/ of both. Three-phase TM images of 1990, 2000 and 2010 from free download data of U.S. Geological Survey (http://glovis.usgs.gov/) were used.

LUCC directly reflects the intensity of the impact of human activities. The influence of LUCC on hydrological processes will directly lead to changes in water supply and demand relations. Thus, exploring the effects of LUCC on karst hydrology and water resources is the key point to alleviate the contradiction between human activities and the natural environment in karst Area. The land cover structure and the change process are analyzed below. Fig 11 shows the land use vector diagram in 1990, 2000 and 2010. Change features of land use since 1990 are shown in Table 2. The main land use types are forestry, grass land and cultivated land, accounting for 99% of the total area. Forest land had the largest proportion among them, taking more than 50% of the total area, followed by dry land, grassland and paddy fields. Due to locational and historical reasons, social development level of karst area in southwest China is low. And the rural population is heavy and the economic development depends heavily on the land resources [58]. Liudong River Basin is no exception, and the proportion of cultivated land accounted for more than 30% during 1990 ~2000.

**Table 2:** Area and proportion of land use during 1990–2010.

Land Use	1990		2000		2010	
	Area/km^2^	Rate/%	Area/km^2^	Rate/%	Area/km^2^	Rate/%
Forest	758.32	50.72	754.31	50.41	822.47	54.96
Grass	247.32	16.54	239.77	16.02	417.36	27.89
Dry Farmland	275.72	18.44	270.31	18.06	137.62	9.20
Paddy Field	203.54	13.61	220.26	14.72	107.53	7.19
Water	7.89	0.53	8.04	0.54	7.05	0.47
Building Lot	2.42	0.16	3.76	0.25	4.45	0.30

**Fig 12:**
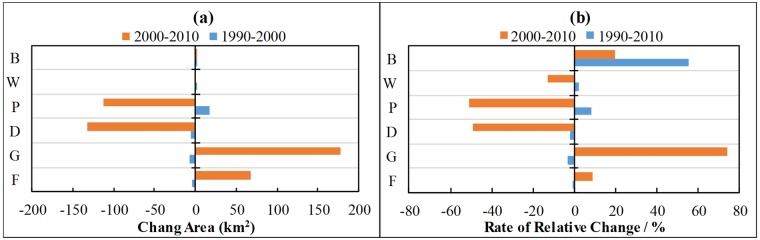
Amount of landuse change (a) and rate of relative change of landuse (b) in different stages. Blue and red bars respectively represent period 1990–2000 and 2000–2010. In this figure, F, G, D, P, W, B are short for forest, grass, dry farmland, paddy field, water and building lot, respectively.

Fig 12 shows landuse change in the different stages. From 1990 to 2000, forestry, grass land and arid land showed no significant decrease. The paddy field increased by 1.11%, and the relative change rate of building lot was 55.17%. The land use change features in this period were rapid expansion of building lot, no significant increase of cultivated land area and no great change of overall land cover. From 2000 to 2010, forestry and grass land increased by 4.55% and 11.87%, respectively, and the relative rate of increase of grass land reached 74%. Arid land and paddy field decreased by 8.86% and 7.53%, respectively, and showed approximately 50% relative rate of decrease. The building lot showed a continuous increasing trend. The water area slightly decreased. The land use change features in this period were significant increase of natural forest and grass land. In particular, grass land increased significantly. The cultivated land decreased greatly, and the building lot increased continuously.

**Fig 13:**
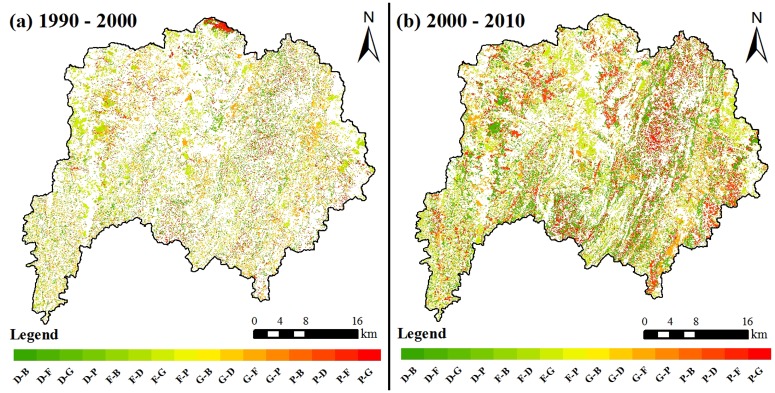
Land cover changes through 1990–2000 (a) and 2000–2010 (b) in study area. F, G, D, P, W, B are short for forest, grass, dry farmland, paddy field, water and building lot, respectively. Maps in Fig 13 were generated by building land use transition matrix using ArcGIS 10.2 (the URL link is http://www.esri.com/) based on three-phase land use vector diagram which generated in Fig 11.

The transition matrix of each land use type during different time periods are shown in Fig 13 and Table 3. From 1990 to 2000, the overall performance of forestry was the mutual transition between grass land and cultivated land, and no significant change in total area existed. Moreover, 45% of the area transited from grass land formed cultivated land, and the area increment of paddy field mainly came from the transition of grass land and arid land. The expansion of building lot was mainly composed of cultivated land transition, which accounted for 89.5%. From 2000 to 2010, the area increment of forestry and grass land mainly came from cultivated land transition, in which the arid land transition was significant. A part of paddy field was transited to arid land. The expansion of building lot was mainly composed of cultivated land transition. In summary, the land use change in the drainage basin was featured with mutual transition between grass land and cultivated land and continuous expansion of building lot since 1990. From 1990 to 2000, the grass land and cultivated land transited mutually. However, no significant change in the total proportion occurred. From 2000 to 2007, the cultivated land transited to grass land in great proportion, and a part of paddy field transited to arid land.

**Table 3:** Land use transition matrix between 1990-2010(%).

Periods	Land Use	Forest Land	Grassland	Dry Farmland	Paddy Field	Water	Building Lot
1990–2000	Forest Land	82.96	9.96	4.67	2.39	0.00	0.01
	Grassland	25.71	54.41	16.02	3.83	0.01	0.02
	Dry Farmland	13.14	8.37	61.16	17.15	0.02	0.17
	Paddy Field	12.26	3.15	12.79	71.30	0.05	0.45
2000–2010	Forest Land	77.44	19.69	1.47	1.39	-	0.01
	Grassland	45.18	48.89	4.56	1.37	-	-
	Dry Farmland	33.34	39.81	18.09	8.69	-	0.07
	Paddy Field	17.92	19.79	30.26	31.82	0.01	0.21

In general, the cultivated land area in the basin has decreased since 1990, especially into the 21st century. Forest land, grassland and residential area were increased and the regional ecological environment had improved remarkably. These were concerned with resources and preferential policies the state invested to promote the development of the karst areas in the last ten years [58].

Before the 1980s, forestry and grass land area in the research region were significantly damaged under the influence of historical events in the previous period[59], such as the Great Leap Forward, Mass Movement to Produce Iron and the influence of agriculture and economic reform, which made the proportion of cultivated land accounted for more than 30% during 1990 ~2000. Since the 1980s, China has implemented several large-scale ecological construction projects in Guizhou Province. By the end of 2004, the comprehensive control area of soil and water conservation achieved 22230.15 km^2^ in the entire province, which accounted for 12.6% of the total area in the entire province [60]. From 1987 to 2000, the soil and water loss area in the province decreased by approximately 5%. According to the strength variation of soil and water loss, the slight and medium loss areas increased by 9.22% and 8.43%, respectively, and the high degree and extremely high degree of soil and water loss areas decreased by 46.37% and 57.64%, respectively [60]. Measures of conversion of cropland to forest and closed forest in the study area led to an increase of green and decrease of cultivated land. And the regional ecological environment has improved gradually. Since the 2000s, some construction projects, such as key project control of soil and water conservation and small drainage basin control, were strengthened in the whole province. And since 2006, the comprehensive control of rock desertification has been fully implemented in the research region [61,62]. The forest and grassland areas in the research region increased greatly in 2010 compared with 2000. The cultivated land, particularly for grass land, decreased greatly, and the ecological environment was in a good restoration condition.

The superposition of these human activities in a short period had led to a rapid change of surface cover, water resources and land use patterns of karst areas, and further triggered the karst hydrological system changes and response.

#### 3.2.2. Analysis of human activity influence

**Fig 14:**
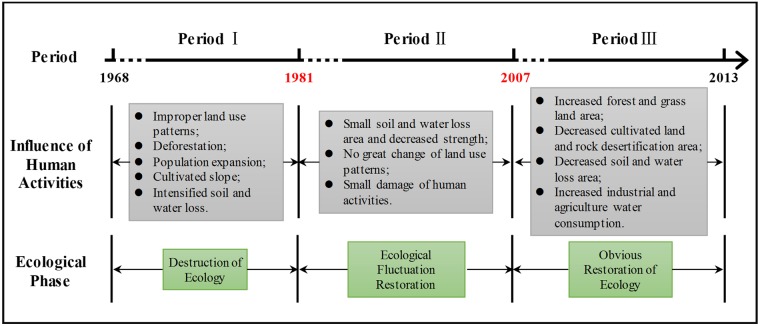
The time axis of influence of human activities during the research period. Basis for division of periods are the same as Fig 10. Content in gray squares indicate the main human activities of different stages. Content in green squares show ecological phase of different stages.

The driving factors of the three-stage change are analysed for the runoff process in this drainage basin according to the time axis of the main human activities during the research period (Fig 14) and the space–time variation analysis of land use during each period mentioned earlier.

From 1968 to 1980, the improper land use patterns and vegetation destruction increased the total runoff and intensification of fluctuation amplitude. Relevant studies have shown that the surface runoff in karst regions is difficult to produce. However, if surface vegetation is greatly interfered and damaged by human activities, then the rain water intercepting, collecting and holding capacity of vegetation are continuously impaired. The coefficient of surface runoff increases in a mutation type when the precipitation reaches a certain level [46]. The research conducted by Huang et al. (2012) [63] showed that, once the forest is regressed to grass–shrub slope and other vegetation after suffering damages, a fold increase of surface runoff occurs, thereby deteriorating the surrounding ecological environment. Therefore, the decrease of forest coverage and the increase of bedrock exposure led to serious soil and water loss in the study area, and the speed and intensity of water eco-environmental degradation intensified rapidly.

Soil and water conservation policy was implemented fully in the research region from 1981 to 2006. The drainage basin entered ecological restoration period, and precipitation played a leading role in runoff variation. Human activity interference had a limited influence on the annual runoff in the research region in this period.

Since 2007, the runoff fluctuation amplitude has become considerably smaller than the precipitation variation because of the obvious restoration of ecology and abrupt increase in human water consumption. On the one hand, Ecological restoration projects strengthened the water conservation capacity of the watershed. Li et al. (2009) [45] reported that the difference in annual runoff amongst different vegetation types in a typical karst region is obvious. Runoff in agriculture land was two to three times of that in shrub forest. However, forest land converted from cultivated land showed good soil and water conservation effectiveness. Grassland demonstrated a strong detention effect on rainfall at a small rainfall intensity. The research on karst region conducted by Li et al. (2012) [64] also showed that forest and grassland, that elicit a good detention effect on water, increase the infiltration of runoff in soil and surface leakage, which greatly decreases runoff. Therefore, the effective control of soil and water loss and ecological restoration in this stage increased the function of water storage and retention of vegetation and soil in the study area, resulting in an increase in runoff at low water level and a decrease in runoff at high water level [65]. So the fluctuation of runoff was mitigated significantly. On the other hand, economic development and population growth have increased sharply the demand for water resources, which directly decreased river runoff amount. Total water consumption in Guizhou Province from 1999 to 2013 showed a gradual increasing trend from 3.833 billion m^3^ in 1999 to 4.507 billion m^3^ in 2013 [60]. The increase in industrial water consumption was the lead cause of the increase in total water consumption. Since the 2010s, the utilisation rate of water resources in Zhujiang drainage basin in Guizhou Province has exceeded 10%, which was obviously higher than that at the beginning of the 21st century.

### 3.3. Quantitative assessment of relative impacts of climate change and human activities on runoff trends

The trend analysis can only give a qualitative description of the impact of climatic factors and human activities on runoff changes. Their contributions will be calculated quantitatively by double-cumulative curve method below. According to the above analysis, there is no obvious correlation existed between the annual runoff and the annual average temperature in the typical karst region in southwest China in the recent 50 years and that the evaporation capacity exerted minimal influence on runoff variation. Meanwhile, the influence of temperature on runoff is considerably smaller than that of precipitation and other human activities because the short-term temperature fluctuation is usually small. Hereby, climate factors which influence runoff variation in the research region are simplified as precipitation, and non-natural factors are summed up as human activities. Human activities influence various fields, including changing underlying surface, implementation of soil and water conservation measures, water consumption variation and so on. The causes of land use variation are difficult to analyse because the annual variation values of subitem land use in this drainage basin are difficult to collect, and some statistics data are not true. Thus, only the contribution rate of comprehensive influence of human activities on runoff variation is discussed in this article.

**Fig 15:**
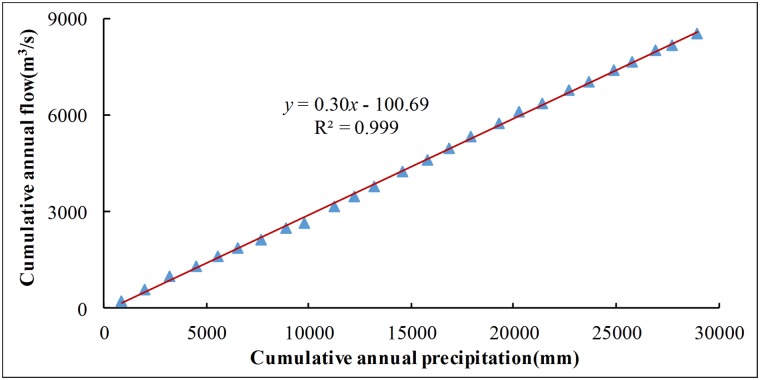
Double mass curve of annual precipitation and runoff during 1981–2006. Distribution of blue triangles show cumulative values of annual precipitation and runoff. Red line indicates the regression curve line of accumulated precipitation and the accumulated runoff that establishing by linear fitting.

The regression prediction equation is obtained by establishing the regression relationship between the accumulated precipitation and the accumulated runoff in the reference section (Fig 15). The mathematical formula is expressed as: *y = 0*.*299x-100*.*69*, where *R*^*2*^ *= 0*.*999* and *P<0*.*001*. The equation has a good fitting degree. Therefore, this regression equation can be used to restore the runoff in the variation section to the condition of the time period when human activities exert a small influence on runoff.

**Fig 16:**
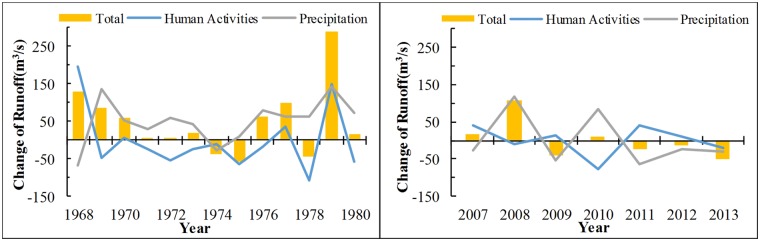
The influence of human activities and climate change on runoff in variation section. Yellow bars indicate the total runoff difference between the reference and variation sections. Blue and gray lines show the runoff variations caused by human activities and climate change, respectively, that were calculated according to Formulas (10) and (11). Criteria for the division of the reference section and variation section is the same as Fig 10.

The influence of human activities and climate change on runoff is calculated according to Formulas (10) and (11). Fig 16 shows the result. Except the precipitation during 1968 and 1974, precipitation generally exerted a mobilisation effect on runoff variation. In 1968, the mobilisation effect of human activities balanced the decreasing effect of precipitation and greatly increased runoff. From 1969 to 1976, the influences of human activities on runoff in the drainage basin played a negative role and reduced the flow. From 1977 to 1980, the influence of human activities on runoff variation alternatively played positive and negative roles, thereby greatly intensifying the fluctuation amplitude of runoff. Except those in 2013, the effects of human activities and precipitation from 2007 to 2013 were opposite and balanced mutually, thereby greatly decreasing the fluctuation amplitude of runoff. This result is consistent with the preceding analysis, which shows that the influence of human activities on runoff is inconsistent in different years.

Table 4 shows the statistical table of contribution rates of human activity interference and climate change to runoff variation in the research region from 1968 to 1980 and from 2007 to 2013. In the variation section from 1968 to 1980, the increasing and decreasing influences of human activity interference on the runoff in the research region appeared alternatively. The average value was −81%, whereas the average value of contribution rate of climate change was 181%. In the variation section from 2007 to 2013, the average value of influence of the significant increase of forest and grass area and the great increase of water consumption on runoff variation was −117%, and the contribution rate of climate change was 217%.

**Table 4:** The impact of climate change and human activities.

Year	Change of Runoff (m^3^/s)	Impacts of Human Activities/%	Impacts of Precipitation/%	Year	Change of Runoff (m^3^/s)	Impacts of Human Activities/%	Impacts of Precipitation/%
1968	130.91	151	-51	1978	-42.70	249	-149
1969	87.02	-55	155	1979	290.76	51	49
1970	58.41	10	90	1980	14.90	-384	484
1971	7.69	-300	400	2007	15.73	260	-160
1972	6.66	-809	909	2008	107.86	-9	109
1973	19.03	-118	218	2009	-41.28	-33	133
1974	-36.31	27	73	2010	9.42	-799	899
1975	-55.92	114	-14	2011	-21.99	-185	285
1976	62.70	-27	127	2012	-11.66	-89	189
1977	99.31	36	64	2013	-49.86	39	61

According to the preceding analysis, the precipitation variation during the research period still controls the runoff evolution trend to a great extent. However, human activities also exert a strong influence on the variation law of runoff.

In this study, we investigate the contribution rate of human activities and climate change to runoff variation but not for the overall fluctuation of runoff. The double mass curve is applied in this study to calculate the contribution rates of human activities and climate change to runoff variation. However, this method finally overestimates the contribution rate of human activities and underestimates the contribution rate of climate change when separating the hydrological effect of these two elements mainly because the hydrological effect of precipitation and nonprecipitation element is separated by this method. Precipitation is the decisive factor affecting runoff variation. However, the temperature rise increases evaporation and decreases runoff. Although the results of the previous analysis indicated that the temperature in the study area has no significant effect on runoff, the possible effect of temperature increase is hard to take into consideration in this study. Therefore, the estimated influence of human activities on the runoff in this research is the maximum possible contribution rate. Nevertheless, the temperature change amplitude in the short term is usually small, and its influence on runoff is considerably smaller than that of precipitation and other human activities. Therefore, the evaluation result obtained in this study is basically accurate and has an important reference value.

## 4. Conclusions

In this study, the following conclusions are obtained by discussing the evolution trend and variation period of runoff and precipitation in the drainage basin through linear fitting, Morlet wavelet analysis, normalized curve and double mass curve with the runoff and precipitation data in the recent 50 years and by analysing the response of runoff to human activities and climate change by establishing precipitation–runoff model:

- Runoff in the karst watershed during the research period exhibits a three-stage change. The abrupt change points are the years 1981 and 2007. The first stage is 1968–1980. During this period, the runoff initially exhibited a trend of sustained decreasing and then an abrupt fluctuation. Improper land utilisation and serious forest and grass destruction intensified the fluctuation variation amplitude of the runoff. The second stage is 1981–2006. During this period, the variation curves of runoff and precipitation exhibited good synchronism. Precipitation significantly affected runoff variation and human activities had a small interference degree. The third stage is 2007–2013. During this period, the fluctuation range of runoff was considerably smaller than that of precipitation. The significant growth of forest and grassland areas and the increase in water consumption mitigated runoff fluctuation and greatly diminished runoff variation amplitude.
- The influences of precipitation and human activities on runoff are separated by applying double mass curve. The contribution rates of human activities and precipitation to runoff variation from 1968 to 1980 were −81% and 181%, respectively. The average contribution rates of human activities and precipitation to runoff variation from 2007 to 2013 were −117% and 217%, respectively.
